# Supplementary material for: Impact of 1-Hour Bundle Achievement in Septic Shock
Source: J Clin Med. 2021 Feb 2;10(3):527. doi: 10.3390/jcm10030527 (PMC7867161; doi:10.3390/jcm10030527)
Supplement: Supplementary file 1 [file jcm-10-00527-s001.pdf]

Table S1. Global univariate logistic regression analysis for in-hospital mortality.

**Variables**	**Unadjusted OR**	**95% CI of OR**	***p* value**
Age, year	1.028	1.017-1.038	<0.001
Male	1.415	1.090-1.838	0.009
SBP, mm Hg	1.000	0.995-1.004	0.891
DBP, mm Hg	0.998	0.991-1.005	0.574
Heart rate, beat per min	1.005	1.000-1.010	0.064
Respiratory rate, per min	1.074	1.050-1.098	<0.001
Body temperature, °C	0.663	0.598-0.734	<0.001
Comorbidities			
Hypertension	1.018	0.786-1.319	0.891
Diabetes mellitus	0.970	0.727-1.294	0.834
Cardiac disease	1.108	0.770-1.595	0.579
COPD	1.403	0.894-2.202	0.140
CKD	1.516	0.970-2.369	0.068
Chronic liver disease	1.219	0.824-1.802	0.322
Infection site			
Respiratory	2.233	1.699-2.936	<0.001
Urinary tract	0.338	0.225-0.509	<0.001
Gastrointestinal tract	1.259	0.884-1.793	0.202
Hepato-biliary & pancreas	0.674	0.464-0.978	0.037
Others	0.493	0.235-1.036	0.062
Lactate, mmol/L	1.291	1.238-1.346	<0.001
SOFA	1.236	1.183-1.290	<0.001
APACHE 2	1.083	1.068-1.098	<0.001
Positive blood culture	1.019	0.791-1.314	0.883

APACHE 2: Acute Physiology and Chronic Health Evaluation 2, CI: confidence interval, CKD: chronic kidney disease, COPD: chronic obstructive pulmonary disease, DBP: diastolic blood pressure, OR: odds ratio, SOFA: Sequential Organ Failure Assessment.

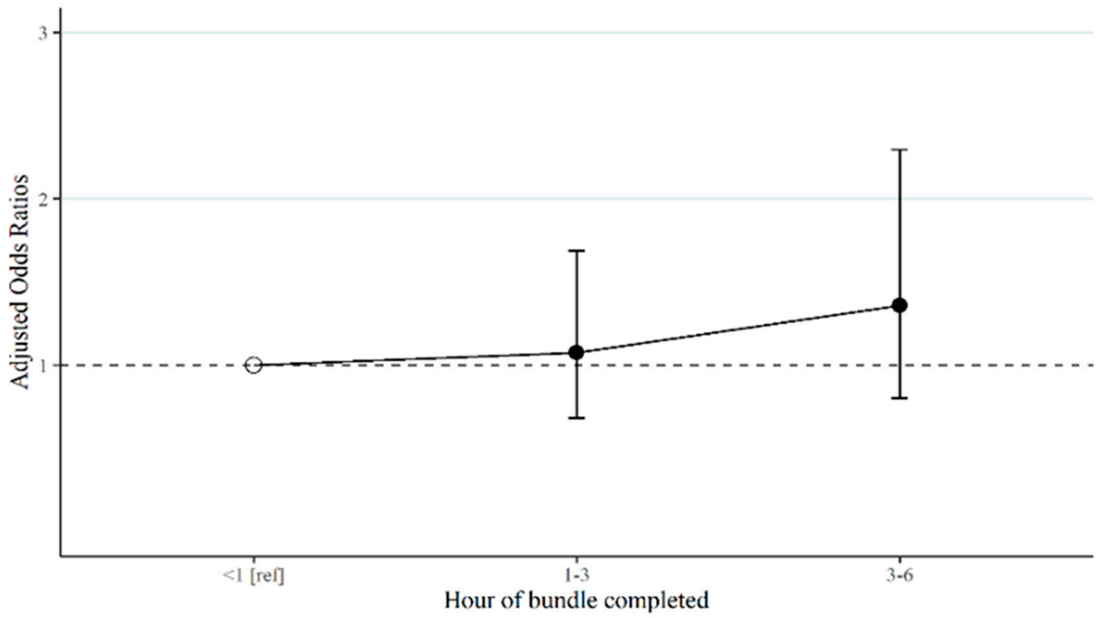

Group	Adjusted odds ratio	Confidence Interval (lower)	Confidence interval (higher)	p-value
<1 vs 1-3	1.075	0.684	1.689	0.721
<1 vs 3-6	1.358	0.803	2.297	0.191

Figure S1: Adjusted odds ratios of each group for in-hospital mortality over time (≤1 h; reference) in multivariable logistic regression analysis.

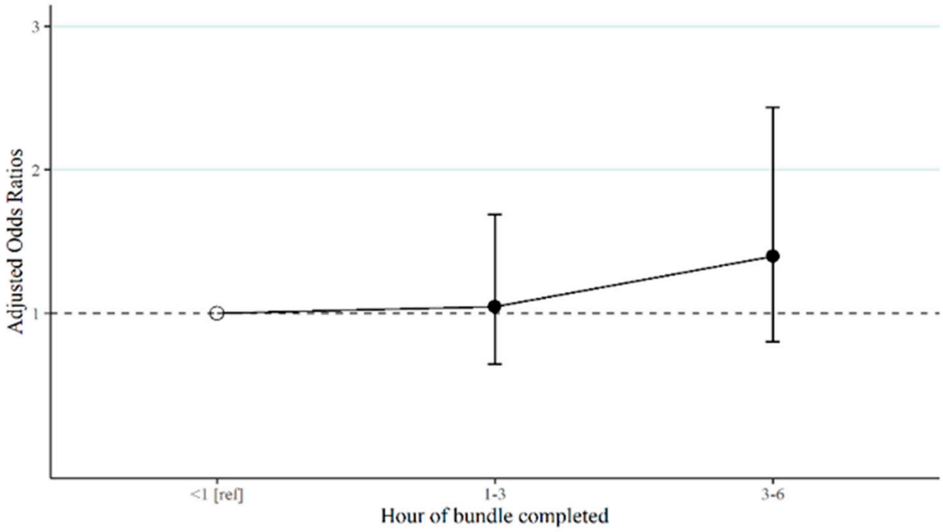

Group	Adjusted odds ratio	Confidence interval (lower)	Confidence interval (higher)	p-value
<1 vs 1-3	1.045	0.647	1.688	0.836
<1 vs 3-6	1.397	0.801	2.435	0.177

Figure S2: Adjusted odds ratios of each group for 28-day mortality over time (≤1 h; reference) in multivariable logistic regression

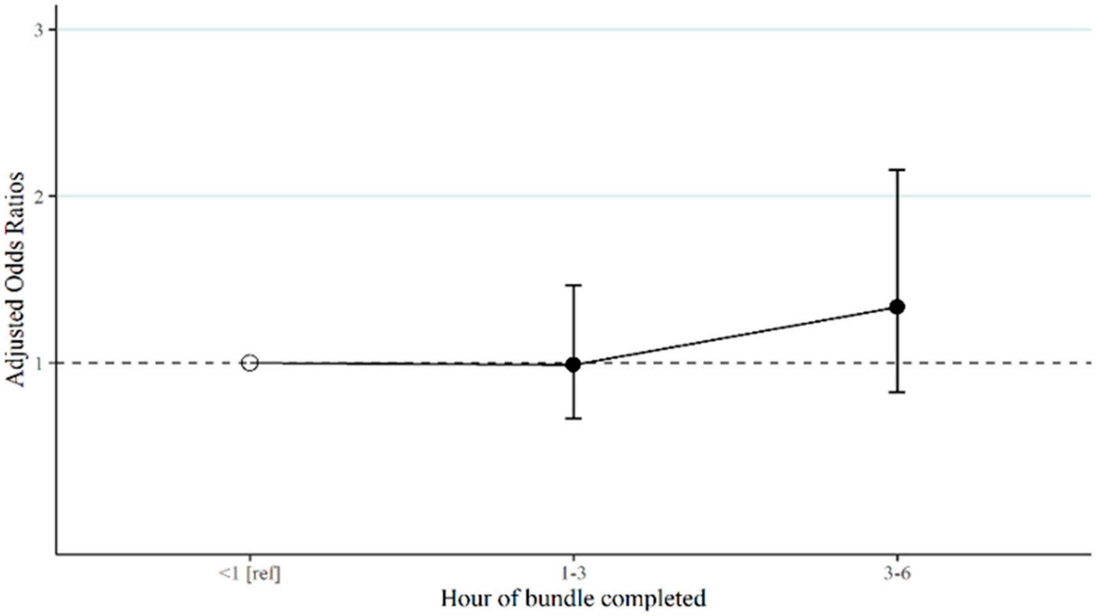

Group	Adjusted odds ratio	Confidence interval (lower)	Confidence interval (higher)	p-value
<1 vs 1-3	0.988	0.666	1.467	0.946
<1 vs 3-6	1.335	0.826	2.157	0.177

Figure S3. Adjusted odds ratios of each group for 90-day mortality over time (≤1 h; reference) in multivariable logistic regression analysis.
